# Surgical Diseases in North Korea: An Overview of North Korean Medical Journals

**DOI:** 10.3390/ijerph17249346

**Published:** 2020-12-14

**Authors:** Sejin Choi, Taehoon Kim, Soyoung Choi, Hee Young Shin

**Affiliations:** 1 Department of Translational Medicine, Seoul National University College of Medicine, Seoul 03080, Korea; peter8909@snu.ac.kr; 2 Seoul National University College of Medicine, Seoul 03080, Korea; taehoon.kim@snu.ac.kr; 3 Institute for Health and Unification Studies, Seoul National University College of Medicine, Seoul 03087, Korea; thdud31@snu.ac.kr; 4 The Republic of Korea National Red Cross, Seoul 04629, Korea

**Keywords:** North Korea, surgery, non-communicable diseases, global health

## Abstract

Information regarding surgical diseases in North Korea is not fully reported despite their clear clinical significance. The current study aimed to indirectly assess the contemporary research trends and medical infrastructure related to surgical diseases in North Korea. We analyzed and categorized articles from the journal *Surgery* that were published during the Kim Jong-un era (2012–2018). The framework for categorization was primarily based on disease entities, surgical specialty, and research methodology. A total of 1792 articles in 28 issues were included in the current study. The frequency of detailed surgical cases and their associated characteristics based on their specialty were investigated. The types of medical imaging techniques and anesthetics that were commonly utilized in clinical fields in North Korea were also evaluated. A large proportion of essential surgeries were covered, with the exception of those for congenital diseases; however, a lack of surgical techniques and infrastructure was revealed. Going forward, further evaluation of the surgical system and a greater focus on non-communicable diseases in North Korea are needed.

## 1. Introduction

In general, information on the health and disease status of 24 million North Koreans is largely unknown due to limited access to the country itself [1]. Only a small number of people have witnessed North Korean healthcare, which is primarily confined to the capital city of North Korea, Pyongyang. Data sources are scarce and gathering reliable data has long been a challenge [2,3]. Additionally, there are a limited number of peer-reviewed articles written in English by North Korean medical researchers, confounding the ability to capture the full picture. 

Some limited facts are known regarding the health of individuals in North Korea, including the average life expectancy of 71.69 years as of 2016 and the number one cause of death, which is cardiovascular disease [2,4]. However, details remain missing and the picture is incomplete. The limited reports available about North Korea have disproportionately focused on nutritional issues and communicable diseases, including parasitic diseases and tuberculosis [5,6,7,8,9]. 

In an attempt to gather additional data, there have been efforts to investigate defectors from North Korea rather than current residents [10]. Furthermore, efforts have been made to examine reports in North Korean medical journals in the fields of internal medicine, pediatrics, obstetrics and gynecology, and psychiatry [11,12,13,14]. These attempts have helped to clarify the details of North Korea’s current medical needs, including the importance of issues such as non-communicable diseases, which were previously underestimated [15].

Notably, there are few reports regarding the status of surgical diseases and surgical environments in North Korea [16]. Regardless of the scarcity of information, the surgery needs are clear, particularly in the case of reducing maternal/child mortality, lightening the burden of trauma, and treatment for life-threatening congenital anomalies, as emphasized in the WHO Global Initiative for Emergency and Essential Surgical Care. 

According to the Institute for Health Metrics and Evaluation, the top 11 causes of death in North Korea are stroke, chronic obstructive pulmonary disease (COPD), ischemic heart disease, liver cancer, lung cancer, Alzheimer’s disease, stomach cancer, road injuries, lower respiratory infection, hypertensive heart disease, and cirrhosis [17]. Among these, five disease categories (hemorrhagic stroke, liver cancer, lung cancer, stomach cancer, road injuries) require surgery to reduce mortality or morbidity and increase the chances of survival. Providing surgical treatment can be highly cost-effective, and a clear picture of the burden of surgical diseases in North Korea can help high-income countries prioritize the dispatch of appropriate medical aid to North Korea [18,19]. 

In the current study, medical research pertaining to surgical diseases in North Korea was investigated through the North Korean medical journal *Surgery*. A secondary aim of the present study was to indirectly assess the nature of surgical diseases and the status of infrastructures in North Korea for surgeries, including anesthesia and radiological imaging.

## 2. Materials and Methods 

All articles published in the North Korean Journal *Surgery* [*Oegwa*] from 2012 to 2018 were reviewed. The selection of articles began in 2012 to correspond to the year when Kim Jong-un became the Chairman of the Workers’ Party of North Korea. The journal *Surgery* was selected as a representative medical journal to understand surgical diseases in North Korea out of nine known medical journals in North Korea: *Internal Medicine*; *Surgery*; *Basic Medicine*; *Korean Medicine*; *Preventive Medicine*; *Pediatrics, Obstetrics, and Gynecology*; *Dentistry, Ophthalmology, Otorhinolaryngology*; *Korean Pharmacy*; *Medical Science* [20]. Obstetrical, gynecological, dental, ophthalmological, and otorhinolaryngological surgeries were excluded from the analysis. These journals are published by Korea Science & Encyclopedia Publishing House or the Central Information Agency for Science and Technology, but it is not known whether these are peer-reviewed [20]. The journal issues were obtained from the Ministry of Unification’s “North Korea Resource Center” in Seoul, South Korea. The articles were reviewed and analyzed by three reviewers, which included a medical doctor (Sejin Choi), an expert on North Korea (Soyoung Choi), and a medical student (Taehoon Kim). As this research is a retrospective review of previously published articles, it was not appropriate or possible to involve patients or the public in the design, conduct, reporting, or dissemination plans. 

Based on a prior study, disease entities were categorized into communicable, non-communicable, maternal, neonatal, nutritional, injury/trauma, and other [21]. Surgical specialties were classified based on the categories introduced by the American College of Surgeons, as follows: general surgery, cardiovascular and thoracic surgery, obstetrics and gynecology, neurological surgery, ophthalmic surgery, oral maxillofacial surgery, orthopedic surgery, otorhinolaryngology, urology, plastic and reconstructive surgery, rehabilitation medicine, and anesthesiology [22].

The research methodology for each article was identified and categorized as follows: observational, randomized controlled trial (RCT), prospective cohort, retrospective cohort, case-control, case report or series, basic science or experimental, systematic review, meta-analysis, level 5 (editorial, educational, letter, perspective), non-systematic review of literature, qualitative paper, mixed study, or other. 

## 3. Results

### 3.1. Structure

In the North Korean journal *Surgery* [*Oegwa*], between 2012 and 2018, an issue was published every quarter with an average of 64 articles per issue. A total of 1792 articles in 28 issues were published from 2012 to 2018. All of the articles were written in Korean and could be classified into seven different types: editorial, original article, discussion, experience, review article, case report or series, or creative invention. The distribution of article types is reported in Table 1. Approximately 60% of articles were original articles, and approximately 15% were case reports. The “creative invention” section was newly added in 2018 to introduce original medical devices.

The majority of original articles reviewed were organized in sections composed of a gyo-si (a quote by the Chairman, optional), study population, study method, study results, conclusion, references, and keywords. “Discussion” articles had similar structures but did not include references. Articles categorized as “experiences” similarly had a structure that consisted of sections including population, methods, results, and conclusion, without any references. 

Out of the 1792 articles, approximately 36% had teachings from Kim Jong-il or Kim Jong-un as a gyo-si (see Table S1). Teachings from Kim Jong-un first appeared on two different occasions in 2018. The contents of these quotes were not directly linked to the topic of the article. Medical images, such as X-ray, CT, or MRI images were not included in any articles. 

### 3.2. Analysis by Disease Category

The results of the analysis by disease category are shown in Table 2. In total, 25.7% of the journal articles that covered surgical diseases fit into the trauma category; 4.6% into the neonatal, pediatric, or congenital disease categories; 35.2% into the non-communicable diseases category. There was no overlap between categories, and there was only one article categorized as maternal disease covering post-operative complications after C-sections. 

### 3.3. Analysis by Disease Subspecialty

The distribution of articles by specialty is demonstrated in Table 3. From 2012 to 2018, 29.6% of the articles were written about diseases within the orthopedic surgery specialty, and 29.5% within the general surgery specialty. Other specialties included in the research articles were categorized as cardiothoracic surgery (8.6%), plastic and reconstructive surgery (7.5%), and neurological surgery (6.2%).

#### 3.3.1. General Surgery

General surgery included those on the lower and upper gastrointestinal (GI) tract, hepatobiliary system, vascular system, thyroid, and breast.

- Trauma: There were a total of six articles categorized as trauma, which included a splenic injury (2 articles), liver injury (2 articles), and traumatic hemorrhagic shock (2 articles).
- Pediatric: In terms of pediatric surgery, between 2012 and 2018, the articles covered intussusception (5 articles), hernia (4 articles), biliary ectasia (3 articles), megacolon (2 articles), hypertrophic pyloric stenosis (1 article), and Meckel’s diverticulum (1 article).
- Cancer: Papers reported the use of chemotherapy for upper GI cases of advanced gastric cancer. The use of endoscopic surgery for gastric cancer was reported as well. One paper included a population sample of 2483 cases that specifically covered the topic of mucosa-associated lymphoid tissue (MALT) lymphoma, which is highly related to *Helicobacter pylori*. A paper on cholangiocarcinoma included 118 cases, 84 of which were male, and reported that cholangiocarcinoma is most common in individuals in their 50’s in the Democratic People’s Republic of Korea (DPRK). Reports of other cancer-related surgeries included breast cancer (6 cases), which included two on oncoplastic techniques. In terms of rectal cancer, one paper reported the mortality from 1996–2005 as 1.97% for men and 1.34% for women.
- Non-communicable diseases: For lower GI cases, there were 29 papers on hemorrhoids, anal fistula, or anal fissure that included anal surgeries. Between 2012 and 2018, 19 appendicitis/appendectomy cases were reported. The surgical treatment of vascular diseases including thromboangiitis obliterans (TAO), arteriosclerosis obliterans (ASO), and deep vein thrombosis (DVT) was notable.
- Others: There were 21 papers that included the use of laparoscopy. Laparoscopic surgeries were used for appendectomy, cholecystectomy, hernia repair (both pediatric and adult), early gastric cancer, and colectomy surgeries. No cases of laparoscopic lobectomy of the liver or laparoscopic surgeries for the pancreato-biliary system were reported. There were no papers reporting thyroid surgery for either benign or malignant cases.

#### 3.3.2. Orthopedic Surgery

- Trauma: A total of 46.7% (248 out of 531) of orthopedic papers were trauma cases, which included fractures.
- Pediatric: For pediatric cases, there were five papers on developmental dysplasia of the hip (DDH) and three on club foot. In 2015, there was one paper with a study population of 2250 for DDH.
- Cancer: A total of five papers reported on tumors, which included chondroma (1 article), leiomyoma (1 article), osteoma (1 article), and sarcoma (1 article). The additional report included animal models of sarcoma.
- Non-communicable diseases: A total of 27 papers dealt with back pain, including herniated intervertebral disc (HIVD), spinal stenosis, and spondylosis, and an additional nine papers reported on arthropathy or arthritis. Twenty-six papers were on avascular necrosis (AVN), containing both radiological and clinical factors. Seven papers provided cases on synovitis, bursitis, or tendinitis.
- Others: There were six papers that used the 3D finite element method (FEM), six on bone substitutes, and two reporting cases with arthroscopy. Of all the orthopedic papers, 24.7% (131 out of 531) utilized X-ray imaging in their studies, 4.5% (24 out of 531) used CT imaging, and 3.2% (17 out of 531) used MRI (with and without contrast). Novocaine (procaine) was used in 19 papers and the use of lidocaine was found in 11 papers.

#### 3.3.3. Cardiothoracic Surgery

- Trauma: There were six papers on trauma. Traumatic hemothorax, pyopneumothorax, and mitral valve injuries were included, as well as cases of electric injury.
- Pediatric: For pediatric cases, there were three papers on Tetralogy of Fallot, one case report on Marfan syndrome, six papers on ventricular septal defect (VSD), and four on patent ductus arteriosus (PDA).
- Cancer: There were 12 papers on lung cancer and one on a mediastinal tumor. One author (S.R. Ahn) was included in 25% of the papers on lung cancer (3 out of 12). While lung adenocarcinoma was distinguished into tubular, papillary, mucinous, and poorly cohesive type pathologically according to the WHO 2010 classification, the 2015 paper that studied the survival rate of lung adenocarcinoma classified it into mucinous and non-mucinous types.
- Non-communicable diseases: A total of 13 papers addressed problems with valves, of which 76.9% (10 out of 13) were on the mitral valve. Two papers were on pulmonary valves, and the other on aortic valvular disease. There were three papers that examined the treatment of pleuritis, one on pericarditis, and two on pleural effusion. One study reported a case of cardiac tamponade. There were three papers on pneumothorax, two on thoracic sympathectomy, and four on coronary artery bypass grafting (CABG).
- Others: Of all the cardiothoracic papers, 24.7% (38 out of 154) used X-ray imaging, 4.5% (7 out of 154) used CT imaging, and 23.4% (36 out of 154) utilized ultrasonography. There were six papers that used dimedrol, five that used morphine, and four that used novocaine (procaine) for anesthesia.

#### 3.3.4. Neurological Surgery

- Trauma: For cases of epidural hemorrhage (EDH), subdural hemorrhage (SDH), and intracerebral hemorrhage (ICH), except for those involving skull fracture, incidences were higher in men. There were two papers on EDH, nine on SDH, and three on ICH. There was one report of treatment for Parkinsonism caused by CO poisoning.
- Pediatric: There were a total of four congenital disease papers, which included three on moyamoya disease and one on cerebral palsy. There were three papers on adult hydrocephalus, but none on pediatric cases. All hydrocephalus papers used endoscopic third ventriculostomy (ETV) as the surgical method, except for one paper that described the characteristics of normal-pressure hydrocephalus (NPH).
- Cancer: There were 11 papers on brain or spine tumors from 2012 to 2018. For papers addressing brain tumors, two were on CPA tumors, two were on meningioma, and two were on pituitary tumors. In these papers, pituitary tumors were approached using a trans-sphenoidal approach (TSA).
- Non-communicable diseases: Two papers were on myelitis and one paper reviewed the features of ischemic stroke.
- Others: Of the neurological papers, 25.2% (28 out of 111) used CT imaging and 9.9% (11 out of 111) used MRI. Five papers reported using novocaine (procaine).

#### 3.3.5. Plastic and Reconstructive Surgery

Burn and wound treatments were common topics. There were 51 articles (35.2%) covering the topic of burn treatment and 32 (22.1%) on the topic of wounds. In the cases of burn treatment, papers reporting the usage of oriental/herbal medicines (Koryo medicines) were noted. Examples of these treatments included Koryo Burn Ointment, arsenic sulfide, extraction from lilac leaves, egg white–rifampicin mix, *Coptis chinensis*, *Cirsium japonicum* var. *maackii*, and *Lonicera japonica*. Additionally, there was one case report for the treatment of an electric injury. 

Skin flaps were also highlighted in 26 articles (17.9%). A total of four papers were on amputation, three on frostbite, one on scalp laceration, and three papers on pressure sores. 

There were two congenital disease papers, which included case reports of syndactyly and microtia. 

Only two papers utilized X-ray imaging, while a single article examined the clinical usage of pre-operational Doppler sonography on skin flaps. Nine papers reported the usage of novocaine (procaine).

#### 3.3.6. Urology

- Trauma: There were eight trauma papers, among which, five were on traumatic urethral stricture.
- Pediatric: There were six pediatric papers, which included reports on congenital adrenal hyperplasia (CAH), circumcision, phimosis, cryptorchidism, Wilms’ tumor, and hypospadias.
- Cancer: There were 12 papers on urologic cancers. Bladder cancer accounted for half of the articles, while the remaining articles included prostate cancer, cystic cancer, and renal cancer.
- Non-communicable diseases: There were five papers on benign prostate hyperplasia (BPH), one on sparganosis, and 10 papers covering stones in the urinary tract.
- Others: Ten papers utilized ultrasonography and a single paper reported the usage of cystoscopy. Three papers used novocaine (procaine), including a case report of treatment for idiopathic priapism with epidural anesthesia.

### 3.4. Other Characteristics

#### 3.4.1. Reports on Oriental Medicine

The analysis of papers using oriental medicine for surgical diseases is shown in Table S2. The findings indicated that 3.3% of the papers utilized oriental medicine (Koryo medicine) in their research, which may include various types of treatment, such as herbal therapy, acupuncture, physical exercise, or diet [23]. The percentage of papers referencing oriental medicine varied within the published timeframe from 4.6% in 2012 to 1.7% in 2015, but remained under 5% during all the years that were reviewed.

#### 3.4.2. Study Population 

In addition to research on the living human population, studies using cadavers and animals were reported. The proportion of the studies using cadavers or animals is listed in Table S3. Overall, 2% of articles used cadavers, and 6% used animal models. 

#### 3.4.3. Analysis of References 

The distribution and the characteristics of references included in the articles are shown in Tables S4–S6. Approximately 33% of the articles were without references, and the average number of references was 1.38. Out of all references cited, 20% were references published within 5 years of the article. On average, 22.3% of the references were from North Korean journals or textbooks, 12.8% of the references were in Chinese, 2.7% were in Japanese, and 60.7% were in English. From 2012 to 2018, there were nine references (0.4%) in German and 15 references (0.6%) in Russian. Textbooks comprised 76% of all North Korean references and 11% of all English references.

#### 3.4.4. Imaging and Anesthesia 

Details of the usage of medical devices from 2012 to 2018 are shown in Table S7. Approximately 11.9% of papers utilized X-ray imaging, while the reported use of CT imaging or MRI remained under 5% (4.4% and 1.8% each, respectively). Ultrasound was the second most commonly used medical imaging technique listed (9.0%). Articles utilizing single imaging devices accounted for 20.3%, while articles with multiple devices accounted for 5.9% of the publications. A total of 73.8% of papers did not take advantage of any imaging techniques.

For imaging devices, some articles introduced them with specific model names. For example, the EDR-750b, Bulgeungi 66 (Russian product) and F-30III, Tamjeong were used. For endoscopy, the Olympus gif-d2, Intravision-8745, and Model MGA-III were used. For ultrasound, articles reported the use of the Aloka SSD-650 (marketed in 1986 and discontinued in 1997), Aloka-SSD 620 (marketed in 1988 and discontinued in 1996), HP SONOS 5500 2.5MHz (marketed in 1988), ATL Sono-CT HDI-5000 (1996–2006), Medison 128BW, Medison SonoAce 8000, and Sonoace 8800. Additional detail regarding ultrasound machines cannot be specified as these models were manufactured for a long period with different editions [24]. In articles dated between 2012–2014, there were no specific imaging devices described. 

The papers reviewed listed a total of 56 various kinds of injection materials used for anesthesia. The commonly used anesthetics and supplementary medications are listed in Table S8. Novocaine (Procaine) was the most commonly used anesthetic (4.5%). Papers reporting specific medications accounted for 11.6% of overall publications.

#### 3.4.5. Research Methodology

**Figure 1:**
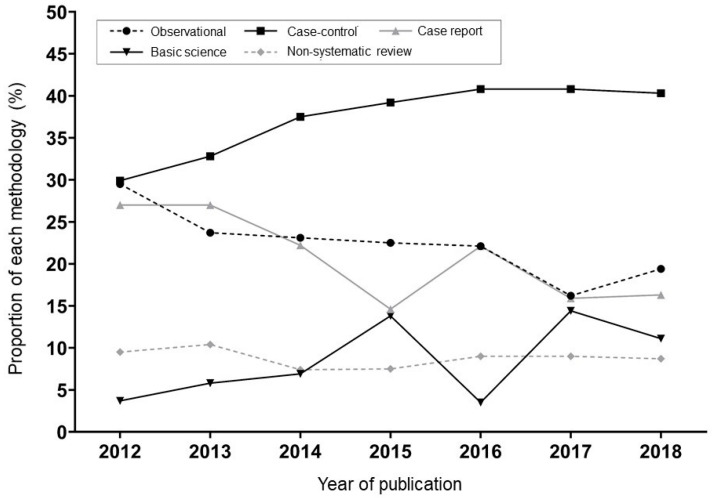
Trends of research methodologies in the journal *Surgery*.

The trends of specific research methodologies are shown in Figure 1. The proportion of case-control studies increased by more than 10%, from 29.9% in 2012 to 40.3% in 2018. The proportion of observational studies and case reports (or series) dramatically decreased (29.5% to 19.4% and 27.0% to 16.3%, respectively). Statistical analysis revealed increasing attention on basic science, as the number of papers on basic science has surged in recent years. Editorial papers with ideological contents, to stress the idea of their supreme leader and elucidate the party line, were consistently published at the beginning of the journals. 

## 4. Discussion

The current study revealed the trends of research conducted in North Korea in the surgical field during the Kim Jong-un era from 2012–2018. An analysis by specialty and disease category reflected the status and characteristics of surgical diseases in North Korea. Trauma had been a constant focus of research, where 25% of articles dealt with traumatic cases that required surgical treatment. Non-communicable diseases were the most common topic, which comprised 35% of journal articles. 

### 4.1. Changes in the Kim Jong-Un Era 

Significant changes have been made in the sector of health and medicine since the beginning of the Kim Jong-un era [25]. The establishment of hospitals, medical research institutes, and factories for medical supplies have contributed to the development of active research programs as authorities attempt to renovate and modernize existing medical facilities [26]. While centralized facilities appear to be the limiting factor for improving the system, the general advancement in medical fields similarly affects the overall quality of surgical medicine and the clinical infrastructure. Researchers and clinicians are establishing preliminary plans to develop medical instruments and introduce up-to-date study outcomes in each clinical field. For example, in 2020, the medical associations organized a committee for plastic surgery [27].

### 4.2. Picture of Surgery in North Korea 

#### 4.2.1. Disease Prevalence and Burden

The high proportion of topics related to non-communicable diseases (NCDs) in the journal *Surgery* (35.2%) supports the idea that the demand for surgery related to NCDs is high in North Korea. NCDs, including tumors, account for eight of the top 10 causes of death in North Korea, among which, stroke, ischemic heart disease, lung cancer, stomach cancer, and cirrhosis may need surgical interventions to reduce mortality and morbidity [17]. Notably, lung cancer and stomach cancer are the fourth and sixth most common causes of death. It is speculated that chronic stress and deterioration in lifestyle and the lack of early intervention led to this outcome [15]. Furthermore, an aging population, a high rate of smoking, and the continuing collapse of the medical system might be other societal contributors [15,28]. The academic interest and demand for NCDs in North Korea may be underestimated and overlooked when considering the articles published in other journals, such as *Naegwa* [*Internal Medicine*], which were not included in the analysis of this study.

The overall trends in South Korea do not differ much from the statistics of North Korea, as lung cancer, liver cancer, stomach cancer, and colorectal cancer are reported to be the third, seventh, ninth, and tenth most common causes of death, respectively [29].

The percentage of articles on the topic of neoplasm among non-communicable diseases were relatively low compared to the actual prevalence. Some types of cancers, such as thyroid cancer, which are reported to be frequent in North Korea, were not reported in any articles between 2012 and 2018 [10]. The steady publication of articles on various types of cancers implies continuous participation and interest in the field, but the lack of pre-/post-operative care, including chemotherapy or radiotherapy and healthcare facilities capable of cancer management, may have resulted in the shortage of research outcomes. There were few if any cases of glioma, though pituitary tumors and use of the trans-sphenoidal approach were published. According to Park and colleagues, the World Federation of Neurosurgical Society (WFNS) delivered a Zeiss Pico Microscope in 2010; therefore, the lack of reports does not solely reflect a shortage of equipment availability [16]. 

Certain surgical treatments, such as liver and kidney transplantation, were not covered in any publications. From this, it can be assumed that organ transplantation is rarely, if ever, performed in North Korea. This could be the result of a lack of surgical techniques or pre-/post-op care, which requires intensive care facilities. 

A high percentage (25% of the total) of articles covered trauma/injury cases ranging from traumatic brain injury accompanied by hemorrhages to fractures and liver or splenic injuries. This may reflect the high number of injuries in North Korea, where road injuries are the fifth most common cause of death [17]. The high incidence of road injuries is similar to that of other developing countries. These findings also coincide with a recent report that pointed out North Korea’s injury rate and inadequate trauma care capacity in detail [30].

#### 4.2.2. Hospital

As the authors’ affiliations were not fully written in the paper, and there was no location information, it is difficult to speculate where these cases were collected. Considering the substandard conditions of provincial hospitals or local clinics in North Korea, it is reasonable to assume that these papers were based on data gathered from tertiary hospitals in Pyongyang [26].

#### 4.2.3. Oriental Medicine

The proportion of papers using oriental medicine (3.3%) was interestingly similar to the proportion of oriental medical doctors (Koryo doctors) among all doctors in North Korea (5.5%; 4384 out of 79,931) [31]. The authorities in North Korea emphasize and encourage the prescription of oriental medicine (Koryo medicine); a number of departments (e.g., Koryo Medicine Production and Management Bureau, Institute of Koryo Medicine) endeavor to manage and develop the usage of oriental medicine [26]. Research outcomes on the utilization of oriental medicine in surgical fields, despite their limited numbers, show distinct characteristics of North Korean surgeries. 

#### 4.2.4. References

One-third of the articles (600 out of 1792) did not include any references, while the proportion of papers with references published in the last five years were below 20%. Furthermore, it was notable that articles from China written in Chinese were frequently cited given that in South Korea, articles written in Chinese are rarely cited in medical journals. A few textbooks or journals were preferred and repeatedly cited; further studies with network analysis on the groups of the references may provide visible relationships. 

#### 4.2.5. Imaging and Anesthesia

In terms of imaging, there were 78 papers (4.4%) that mentioned CT imaging in the study method and 33 (1.8%) that reported the use of MRI as an imaging device. Considering these proportions, CT and MRI may not be widely used imaging modalities in North Korea. This also could be a significant hurdle in setting precise surgical plans. 

#### 4.2.6. Medical Devices

Due to sanctions and ongoing political situations, the DPRK has been attempting to develop their medical equipment, including essential spinal implants [16], spiral CT (from Kim Chaek University, issued in 2016), and prosthetic legs made out of unsaturated resin (from Ham Heung, issued in 2016). The production plants are known to be located in Nampo, Hamhung, and Hyangsan; however, the quality and quantity of the supply are not clearly known [32,33]. Modernization of the plants in Hyangsan has been undertaken recently in 2020, producing several types of medical equipment, including operating tables and dental instruments; further studies are needed to figure out how mass production is carried out [33]. Instructions to produce appropriate medical devices are also reflected in the case reports (issued in 2016), with individual attempts to develop new types of devices, such as those for laparoscopic surgery. 

#### 4.2.7. Medical Conferences and Committees

The types of medical conferences held in North Korea are not precisely known, but related comments were made in the journal *Surgery*. In 2017, in issue 1 of the *Surgery* journal, it was stated that the 23rd National Cardiothoracic Conference, 34th National Abdominal Surgery Conference, and 4th Resuscitation Conference were held. From this issue, it can be assumed that regular medical conferences based on specialties are being held in the DPRK. The list of the committees in the North Korean medical association is provided in Table S9 [27]. 

### 4.3. In the Context of Global Surgery 

In 2015, the World Health Organization suggested 44 essential surgeries that are necessary to reduce the burden of surgically avertable deaths worldwide [19,34,35]. Of the 44 essential surgeries, aside from ophthalmology, obstetrics and gynecology, and dental surgeries, 28 were selected for comparison with research from the North Korean Journal *Surgery* (Table 4). Eight surgeries could not be found, including drainage of superficial abscesses, suprapubic cystostomy, or escharotomy. These procedures could have been omitted as they may be too simple and basic to be a focused research topic. However, it is crucial to note that other procedures, such as those for congenital diseases like cleft lip, anorectal malformation, and Hirschsprung’s disease, were also absent from any publications. The need for surgeries related to congenital diseases in North Korea requires further investigation. 

### 4.4. Limitations and Implications

The current study has several limitations. First, this review was conducted using an indirect approach to estimate the epidemiology of DPRK’s surgical needs based on published journal articles as a source of analysis. The research papers reflect North Korean doctors’ research interests and not the full spectrum of disease characteristics or burdens in North Korea. The reviewed research papers did not report where their study populations resided, which could lead to a disproportionate geographical distribution of the study population. A direct survey of the North Korean population, which is almost impossible in the current political climate, is necessary to collect unbiased and reliable data. 

Second, the scope of the analysis only included data from the Kim Jong-un era. A database of previous periods could have given a more complete picture to identify changes in surgical trends. However, the focus on recent periods has its advantages in recognizing the latest efforts in research on surgical diseases and the medical infrastructure. 

Third, articles published in the journal were relatively short in length, with an average of 0.5–1 page per paper. It is possible that the papers included in the journal are not full papers but abstracts.

Fourth, this study only analyzed papers published in the journal *Surgery*; therefore, obstetrical, gynecological, dental, ophthalmological, and otorhinolaryngological surgeries were not mainly covered. Further investigation is needed to grasp the status of these kinds of surgeries. 

Recent research outcomes in North Korea have been published in overseas, peer-reviewed journals under the instruction of Kim Jong-un, which may provide additional information to interpret the current medical situations in one of the most mysterious countries in the world [36]. These surgery-related papers published in international journals were presenting more detailed and well-organized data compared to the relatively short versions of papers in their domestic journal [37,38,39,40]. Furthermore, these were all from Pyongyang Medical College Hospital of Kim Il Sung University and covered topics such as esophageal stricture [40], gastrectomy due to gastric and duodenal ulcers or gastric carcinoma [37], thoracoscopic surgery for hyperhidrosis, Raynaud’s disease, pneumothorax, empyema, lung cancer, mediastinal tumor, and lung tuberculoma [39]. These topics were congruent with what we analyzed in this study. 

Despite these limitations, the insights gained into surgical and medical fields in North Korea from the current study are considerable. The prevalence of surgical diseases may be the key to understanding unmet needs and priorities, and the analysis can be used as guidance for policymakers with a focus on North Korea. The reports of medical imaging techniques and anesthesia use provide a unique perspective to approach and map out future strategies on the utilization of limited medical resources. Further studies on surgical interventions in North Korea may help to identify deficiencies and guide the subsequent necessary steps to rebuild severed relationships. 

## 5. Conclusions

The findings present a preliminary vision of the surgical diseases of North Korea, from the current focus in each surgical specialty to the status of surgical infrastructures. The characteristics of the research trends in North Korea were unique in the Kim Jong-un era; follow-up studies on the betterment in surgical fields may provide fundamental references for policymakers and researchers. Meaningful discussions on North Korean healthcare have, however, been dwindling in recent years; even the remaining part of the attention is disproportionately concentrated on communicable diseases and nutritional issues. Further attention on non-communicable diseases, including surgical diseases, is needed considering the mortality and morbidity burdens. 

## Figures and Tables

**Table 1:** Distribution of article types.

Year	Original Article	Experience	Discussion	Review	Case	Editorial	Invention	Total
**2012**	127 (52.7%)	31 (12.9%)	26 (10.8%)	23 (9.5%)	34 (14.1%)	0 (0.0%)	0 (0.0%)	241
**2013**	132 (54.8%)	21 (8.7%)	18 (7.5%)	25 (10.4%)	44 (18.3%)	1 (0.4%)	0 (0.0%)	241
**2014**	133 (61.6%)	14 (6.5%)	16 (7.4%)	16 (7.4%)	34 (15.7%)	3 (1.4%)	0 (0.0%)	216
**2015**	162 (67.5%)	22 (9.2%)	0 (0.0%)	18 (7.5%)	34 (14.2%)	4 (1.7%)	0 (0.0%)	240
**2016**	165 (57.1%)	26 (9.0%)	24 (8.3%)	26 (9.0%)	44 (15.2%)	4 (1.4%)	0 (0.0%)	289
**2017**	172 (62.1%)	18 (6.5%)	25 (9.0%)	26 (9.4%)	33 (11.9%)	3 (1.1%)	0 (0.0%)	277
**2018**	168 (58.3%)	17 (5.9%)	28 (9.7%)	25 (8.7%)	43 (14.9%)	4 (1.4%)	3 (1.0%)	288
**Total**	1059 (59.1%)	149 (8.3%)	137 (7.6%)	159 (8.9%)	266 (14.8%)	19 (1.1%)	3 (0.2%)	1792

**Table 2:** Articles by disease category.

Year	Trauma	Neonatal, Congenital, Pediatric	Communicable	Non-Communicable	Basic Science	Total
**2012**	53 (22.0%)	14 (5.8%)	3 (1.2%)	79 (32.8%)	20 (8.3%)	169
**2013**	55 (22.8%)	14 (5.8%)	2 (0.8%)	103 (42.7%)	15 (6.2%)	189
**2014**	54 (25.0%)	8 (3.7%)	1 (0.5%)	85 (39.4%)	21 (9.7%)	169
**2015**	58 (24.2%)	12 (5.0%)	2 (0.8%)	90 (37.5%)	18 (7.5%)	180
**2016**	71 (24.6%)	9 (3.1%)	1 (0.3%)	108 (37.4%)	20 (6.9%)	209
**2017**	68 (24.5%)	15 (5.4%)	1 (0.4%)	94 (33.9%)	27 (9.7%)	205
**2018**	101 (35.1%)	10 (3.5%)	0 (0.0%)	72 (25.0%)	22 (7.6%)	205
**Total**	460 (25.7%)	82 (4.6%)	10 (0.6%)	631 (35.2%)	143 (8.0%)	1326

**Table 3:** Distribution of the articles by subspecialty.

Year	NS	OS	PRS	CS	Uro	GS	A	Others	Total
**2012**	16	74	17	25	12	79	12	6	241
**2013**	14	79	12	25	10	80	12	9	241
**2014**	9	61	13	23	13	56	14	27	216
**2015**	19	59	24	16	13	60	21	28	240
**2016**	18	85	27	26	17	79	9	28	289
**2017**	20	68	15	21	9	89	22	33	277
**2018**	15	105	27	19	5	86	19	12	288
**Total**	111 (6.2%)	531 (29.6%)	135 (7.5%)	154 (8.6%)	79 (4.4%)	530 (29.5%)	109 (6.1%)	143 (8.0%)	1792

NS: neurosurgery, OS: orthopedic surgery, PRS: plastic and reconstructive surgery, CS: cardiothoracic surgery, Uro: urology, GS: general surgery, A: anesthesiology.

**Table 4:** Essential surgery topics covered in the journal *Surgery*.

Essential Surgery		Specialty	Covered
General surgical	1. Drainage of a superficial abscess	All	X
	2. Male circumcision	Uro	O
	3. Repair of perforation	GS	O
	4. Appendectomy	GS	O
	5. Bowel obstruction	GS	O
	6. Colostomy	GS	X
	7. Gallbladder disease	GS	O
	8. Hernia	GS	O
	9. Hydrocelectomy	Uro	O
	10. Relief of urinary obstruction: catheterization or suprapubic cystostomy	Uro	O (catheterization) X (suprapubic cystostomy)
Injury	11. Suturing a laceration	All	O
	12. Management of non-displaced fractures	OS	O
	13. Tube thoracostomy	CS	O
	14. Trauma laparotomy	GS	O
	15. Fracture reduction	OS	O
	16. Irrigation and debridement of open fractures	OS	O
	17. Placement of external fixator, use of traction	OS	O
	18. Escharotomy or fasciotomy	PRS	X
	19. Trauma-related amputations	OS	O
	20. Skin grafting	PRS	O
	21. Burr hole	NS	O
Congenital	22. Cleft lip and palate repair	PRS	X
	23. Club foot repair	OS	O
	24. Shunt for hydrocephalus	NS	X (only ETVs mentioned)
	25. Repair of anorectal malformation	GS	X
	26. Hirschsprung’s disease	GS	X
Non-trauma orthopedic	27. Drainage of septic arthritis	OS	O
	28. Debridement of osteomyelitis	OS	O

Uro: urology, GS: general surgery, OS: orthopedic surgery, CS: cardiothoracic surgery, PRS: plastic and reconstructive surgery, NS: neurological surgery, ETVs: endoscopic third ventriculostomies; O: covered, X: not covered.

## Supplementary Materials

The following are available online at https://www.mdpi.com/1660-4601/17/24/9346/s1. Table S1: Citation of remarks (gyo-si) by the Chairman of North Korea. Table S2: Papers using oriental medicine for surgical diseases. Table S3: Papers using cadavers or animals. Table S4: Distribution of references in the articles. Table S5: The languages used in the overall references, Table S6: The languages of the textbooks used as references, Table S7: Medical imaging techniques utilized in North Korean surgery journals. Table S8: Commonly used anesthetics and supplementary medications, Table S9: List of committees in the North Korean Medical Association.
