# Supplementary material for: Surgical Diseases in North Korea: An Overview of North Korean Medical Journals
Source: Int J Environ Res Public Health. 2020 Dec 14;17(24):9346. doi: 10.3390/ijerph17249346 (PMC7764982; doi:10.3390/ijerph17249346)
Supplement: Supplementary file 1 [file ijerph-17-09346-s001.pdf]

Supplementary Materials

Table S1. Citation of remarks (gyo-si) by the Chairman of North Korea.

Year	Kim Jong-il	Kim Jong-un	Total
2012	77	0	77
2013	89	0	89
2014	89	0	89
2015	97	0	97
2016	97	0	97
2017	98	0	98
2018	94	2	96
Total	641	2	643 (35.9%)

Table S2. Papers using oriental medicine for surgical diseases.

Year	Papers with Oriental Medicine	Proportion (%)
2012	11	4.6%
2013	5	2.1%
2014	7	3.2%
2015	4	1.7%
2016	11	3.8%
2017	9	3.2%
2018	12	4.2%
Total	59	3.3%

Table S3. Studies using cadavers or animals.

Year	Cadaver	Proportion (%)	Animals	Proportion (%)
2012	7	2.9%	13	5.4%
2013	3	1.2%	12	5.0%
2014	7	3.2%	14	6.5%
2015	5	2.1%	13	5.4%
2016	2	0.7%	18	6.2%
2017	7	2.5%	20	7.2%
2018	5	1.7%	17	5.9%
Total	36	2.0%	107	6.0%

Table S4. Distribution of references in the articles.

Year	Recent 5-Year References (%)	Average Number of References†		Number of Articles		
		(a)	(b)	with References	without References	Total
2012	25%	1.30	2.17	145	96	241
2013	20%	1.45	2.23	157	84	241
2014	16%	1.50	2.33	139	77	216
2015	20%	1.55	2.16	173	67	240
2016	17%	1.29	1.97	190	99	289
2017	17%	1.38	1.95	196	81	277

2018	15%	1.24	1.86	192	96	288
Total	19%	1.38	2.08	1192	600	1792

†Articles without a reference were (a) included or (b) excluded in the analyses.

Table S5. The languages used in the overall references.

Year	North Korean (%)	Chinese (%)	Japanese (%)	English (%)	German (%)	Russian (%)
2012	63 (20.1%)	52 (16.6%)	5 (1.6%)	186 (59.2%)	1 (0.3%)	2 (0.6%)
2013	81 (23.1%)	63 (18.0%)	5 (1.4%)	194 (55.4%)	2 (0.6%)	2 (0.6%)
2014	67 (20.7%)	54 (16.7%)	10 (3.1%)	192 (59.3%)	0 (0.0%)	1 (0.3%)
2015	94 (25.2%)	44 (11.8%)	3 (0.8%)	226 (60.6%)	0 (0.0%)	5 (1.3%)
2016	94 (25.1%)	25 (6.7%)	25 (6.7%)	219 (58.6%)	4 (1.1%)	2 (0.5%)
2017	86 (22.5%)	43 (11.2%)	9 (2.3%)	240 (62.7%)	2 (0.5%)	3 (0.8%)
2018	68 (19.0%)	36 (10.1%)	9 (2.5%)	245 (68.4%)	0 (0.0%)	0 (0.0%)
**Total**	553 (22.3%)	317 (12.8%)	66 (2.7%)	1502 (60.7%)	9 (0.4%)	15 (0.6%)

Table S6. The languages of textbooks used as references.

Year	North Korean Textbooks	Proportion (%)	English Textbooks	Proportion (%)	Chinese Textbooks	Proportion (%)
2012	45	71%	10	5%	0	0%
2013	59	73%	10	5%	0	0%
2014	48	72%	21	11%	0	0%
2015	65	69%	13	6%	0	0%
2016	70	74%	16	7%	1	4%
2017	65	76%	33	14%	5	12%
2018	52	76%	26	11%	0	0%

The proportions were calculated as follows: the number of textbooks of each language divided by overall number of textbooks.

Table S7. Medical imaging techniques utilized in North Korean surgery journals.

Year	X-ray	CT†	MRI†	Endoscopy	Fluoroscopy	USS	Angiography
2012	22 (9.1%)	11 (4.6%)	7 (2.9%)	3 (1.2%)	7 (2.9%)	18 (7.5%)	2 (0.8%)
2013	33 (13.7%)	13 (5.4%)	7 (2.9%)	2 (0.8%)	6 (2.5%)	31 (12.9%)	4 (1.7%)
2014	28 (13.0%)	5 (2.3%)	2 (0.9%)	6 (2.8%)	4 (1.9%)	15 (6.9%)	1 (0.5%)
2015	21 (8.8%)	8 (3.3%)	5 (2.1%)	9 (3.8%)	12 (5.0%)	20 (8.3%)	0 (0.0%)
2016	35 (12.1%)	14 (4.8%)	2 (0.7%)	7 (2.4%)	11 (3.8%)	31 (10.7%)	1 (0.3%)
2017	34 (12.3%)	12 (4.3%)	4 (1.4%)	7 (2.5%)	7 (2.5%)	21 (7.6%)	4 (1.4%)
2018	41 (14.2%)	15 (5.2%)	6 (2.1%)	4 (1.4%)	12 (4.2%)	25 (8.7%)	5 (1.7%)
Total	214 (11.9%)	78 (4.4%)	33 (1.8%)	38 (2.1%)	59 (3.3%)	161 (9.0%)	17 (0.9%)

† CT and MRI include both cases with or without contrast. CT: computed tomography, MRI: magnetic resonance imaging, USS: ultrasound scan.

Table S8. Commonly used anesthetics and supplementary medications.

Year	Novocaine	Dimedrol	Morphine	Lidocaine	Fentanyl	Atropine	Ketamine	Thiopental	Diazepam
2012	15 (6.2%)	9 (3.7%)	11 (4.6%)	8 (3.3%)	4 (1.7%)	4 (1.7%)	1 (0.4%)	4 (1.7%)	4 (1.7%)
2013	14 (5.8%)	4 (1.7%)	5 (2.1%)	5 (2.1%)	4 (1.7%)	3 (1.2%)	2 (0.8%)	6 (2.5%)	2 (0.8%)
2014	14 (6.5%)	7 (3.2%)	7 (3.2%)	11 (5.1%)	3 (1.4%)	3 (1.4%)	5 (2.3%)	3 (1.4%)	2 (0.9%)
2015	5 (2.1%)	10 (4.2%)	7 (2.9%)	8 (3.3%)	10 (4.2%)	7 (2.9%)	9 (3.8%)	3 (1.3%)	4 (1.7%)
2016	15 (5.2%)	2 (0.7%)	3 (1.0%)	6 (2.1%)	5 (1.7%)	2 (0.7%)	4 (1.4%)	4 (1.4%)	2 (0.7%)
2017	9 (3.2%)	6 (2.2%)	6 (2.2%)	4 (1.4%)	3 (1.1%)	7 (2.5%)	3 (1.1%)	2 (0.7%)	5 (1.8%)
2018	8 (2.8%)	10 (3.5%)	8 (2.8%)	3 (1.0%)	6 (2.1%)	7 (2.4%)	2 (0.7%)	3 (1.0%)	2 (0.7%)
Total	80 (4.5%)	48 (2.7%)	47 (2.6%)	45 (2.5%)	35 (2.0%)	33 (1.8%)	26 (1.5%)	25 (1.4%)	21 (1.2%)

Table S9. List of committees in the North Korean Medical Association.

	Committees	
**Existing**	**Newly Established**	**Planned**
Epidemiology<br>Internal medicine<br>Surgery<br>Koryo medicine<br>(Oriental medicine)<br>Pharmacology<br>**Basic science**	Plastic surgery	Natural treatment †

† Treatments including mineral water, hot springs, and mud.
